# Antigen presenting capacity of murine splenic myeloid cells

**DOI:** 10.1186/s12865-016-0186-4

**Published:** 2017-01-11

**Authors:** Ying-Ying Hey, Benjamin Quah, Helen C. O’Neill

**Affiliations:** 1 Research School of Biology, Australian National University, Canberra, ACT Australia; 2 Clem Jones Research Centre for Regenerative Medicine, Bond University, Gold Coast, Queensland Australia; 3 John Curtin School of Medical Research, Australian National University, Canberra, Australia

**Keywords:** Myeloid cells, Dendritic cells, Antigen presentation/processing, Spleen

## Abstract

**Background:**

The spleen is an important site for hematopoiesis. It supports development of myeloid cells from bone marrow-derived precursors entering from blood. Myeloid subsets in spleen are not well characterised although dendritic cell (DC) subsets are clearly defined in terms of phenotype, development and functional role. Recently a novel dendritic-like cell type in spleen named ‘L-DC’ was distinguished from other known dendritic and myeloid cells by its distinct phenotype and developmental origin. That study also redefined splenic eosinophils as well as resident and inflammatory monocytes in spleen.

**Results:**

L-DC are shown to be distinct from known splenic macrophages and monocyte subsets. Using a new flow cytometric procedure, it has been possible to identify and isolate L-DC in order to assess their functional competence and ability to activate T cells both in vivo and in vitro. L-DC are readily accessible to antigen given intravenously through receptor-mediated endocytosis. They are also capable of CD8^+^ T cell activation through antigen cross presentation, with subsequent induction of cytotoxic effector T cells. L-DC are MHCII^−^ cells and unable to activate CD4^+^ T cells, a property which clearly distinguishes them from conventional DC. The myeloid subsets of resident monocytes, inflammatory monocytes, neutrophils and eosinophils, were found to have varying capacities to take up antigen, but were uniformly unable to activate either CD4^+^ T cells or CD8^+^ T cells.

**Conclusion:**

The results presented here demonstrate that L-DC in spleen are distinct from other myeloid cells in that they can process antigen for CD8^+^ T cell activation and induction of cytotoxic effector function, while both L-DC and myeloid subsets remain unable to activate CD4^+^ T cells. The L-DC subset in spleen is therefore distinct as an antigen presenting cell.

## Background

Spleen is a secondary lymphoid organ that specialises in filtering blood-borne antigen and recycling the heme group from damaged erythrocytes. The spleen is also important for myelopoiesis, and myeloid cells are primarily located within the red pulp region. The white pulp contains the periarteriolar lymphoid sheath in the T-cell zone, B cell follicles and the marginal zone, located at the interface of the red pulp and the periarteriolar lymphoid sheath for screening blood-borne antigens and pathogens [1, 2]. Multiple subsets of dendritic cells (DC) have been described in spleen, located mainly within the white pulp where immune responses against blood-borne antigens and pathogens are initiated.

The “myeloid” subset in spleen includes granulocytes, monocytes and macrophages. Granulocytes like neutrophils, eosinophils, basophils and mast cells are Ly6G^+^ cells mainly localised in the red pulp region, with some cells in transition through the marginal zone into red pulp [3]. Monocytes develop in bone marrow from a common myeloid/dendritic cell progenitor [4, 5], and continuously migrate into blood and spleen as mature cells [6]. When monocytes enter tissues they terminally differentiate to give macrophages, although recent evidence suggests that blood precursors may not be the only source of tissue macrophages, with evidence that they can derive from endogenous progenitors of yolk sac and embryonic origin [7–9].

Two clear subsets of monocytes were originally identified in blood as the CX_3_CR1^lo^Ly6C^hi^ inflammatory monocytes, and the CX_3_CR1^hi^Ly6C^−^ resident monocytes [5, 6]. However, the characterization of monocyte subsets in tissues is still in its infancy, and our own recent investigations suggest that these early phenotypic descriptors do not exactly mirror the phenotype of similar subsets in spleen [10]. Current thinking is that inflammatory monocytes, now sometimes referred to as “classical monocytes” [11], home to sites of infection where they induce an inflammatory response, and may also differentiate to give TNF/iNOS-producing dendritic cells [12]. Resident monocytes are sometimes referred to as “non-classical monocytes” and migrate under steady-state conditions as precursors of tissue-resident macrophages, for example in liver, spleen, lung and skin [11, 13]. However, there are still gaps in our knowledge of the relationship between resident macrophages and resident or non-classical monocytes. Some studies now indicate a multitude of pathways for development, influenced by environmental and infectious states, with a recent evidence for the wound healing capacity of some monocytes. A reservoir of undifferentiated monocytes resident in spleen was found to be similar to blood monocytes in terms of phenotype and gene expression [14]. Monocytes were shown to mobilise from spleen into sites of inflammation in heart, with inflammatory monocytes clearing damaged tissues, and resident monocytes promoting wound healing [14].

Spleen also contains several subsets of resident macrophages. Marginal zone metallophilic macrophages stain with the MOMA-1 antibody and are localised near the PALS and B cell follicles [15]. These macrophages are thought to function in induction of cytotoxic T cell responses against blood-borne and self antigens [15, 16]. Marginal zone macrophages are located closer to red pulp and express a number of Toll-like receptors (TLR), the MARCO scavenger receptor and the C-type lectin, SIGNR1, for clearance of microorganisms [15, 17]. Tangible body macrophages in the white pulp of spleen are involved in phagocytosis of apoptotic B cells during germinal center reactions and can be distinguished as CD11b^−^F4/80^−^CD68^+^ cells [18]. Red pulp macrophages which clear old or damaged red blood cells and recycle of heme groups, are distinct by F4/80 expression (CD11b^+^F4/80^+^CD68^+^) [2]. In addition, macrophages can be further classified as pro-inflammatory (classical) or anti-inflammatory (non-classical) subsets of M1 and M2 macrophages on the basis of functions, adding further functional diversification to the myeloid lineage [19].

Extensive studies on the lineage origin and immune function of splenic DC subsets has led to definition of conventional (c) DC and plasmacytoid (p) DC derived from a common dendritic progenitor in bone marrow [20, 21]. Plasmacytoid-preDC and pre-cDC can then be found in blood [22], and precursors enter and mature in spleen to form three main subsets which activate both CD4^+^ and CD8^+^ T cells [22]. The CD8^+^ and CD8^−^ subsets of cDC are distinct in production pf interleukin-12 and ability to cross -present antigen to CD8^+^ T cells [23], while pDC are strong producers of interferon-α in response to viral infection [24, 25]. However, monocyte-derived DC can also form in spleen in response to inflammatory stimuli [26], and these are distinguishable both marker phenotypically and functionally from cDC [6, 27, 28].

A novel subset of dendritic-like cells, namely L-DC, has also been identified in both murine and human spleen [10, 29, 30]. These cells were discovered on the basis of their resemblance to cells produced in splenic long term cultures [31, 32]. An equivalent L-DC subset in spleen has been identified following comprehensive flow cytometric analysis of many splenic myeloid and dendritic subsets [10]. That study distinguished L-DC from cDC subsets, and served to better define the splenic subsets of inflammatory monocytes, resident monocytes and eosinophils in mice [10]. L-DC are now identifiable by their phenotype as CD11b^hi^CD11c^lo^CD43^lo^CX_3_CR1^lo^ cells, also lacking expression of MHCII, Ly6C, Ly6G, and Siglec-F. They are clearly distinct from inflammatory monocytes, neutrophils and eosinophils on the basis of phenotype and morphology. Inflammatory monocytes were found to be phenotypically and morphologically distinct from resident monocytes, Resident monocytes were found to be more closely related in phenotype to L-DC than to inflammatory monocytes, although all three subsets were morpologically distinct. In order to assess the functional role of L-DC in relation to resident monocytes and other myeloid subsets, pure populations of cells have been sorted for comparison of their antigen presenting capacity.

## Methods

### Animal

C57BL/6J, C57BL/6.Tg(TcraTcrb)1100Mjb (OT-I TCR-transgenic (TCR-tg) (anti-H-2K^b^/OVA_257–264_), C56BL/6. SJL/J.OT-II.CD45.1 (OT-II TCR-tg (anti-IA^b^/OVA_323–339_) mice) and C57BL/6-Tg(CAG-OVA)916Jen:WehiAnu (Act-mOVA) mice were obtained from the John Curtin School of Medical Research at the Australian National University (Canberra, ACT, Australia). Mice were housed and handled according to the guidelines of the Animal Experimentation Ethics Committee at the Australian National University. Mice were euthenased through carbon dioxide asphyxiation.

### Fractionation of cells

Dendritic and myeloid cells were separated from splenocytes via negative depletion of T, B and red blood cells using MACS® magnetic bead technology (Miltenyi: Bergisch Gladbach, Germany). Splenocytes were incubated with red blood cell lysis buffer for 5 minutes and washed with fluorescence activated cell sorting (FACS) buffer (1% FSC, 0.1% sodium azide in Dulbecco’s Modified Eagle Medium). Cells were then stained with 0.25 μg biotinylated anti-Thy1.2 antibody/10^8^ cells (T cells), 0.25 μg biotinylated anti-CD19 antibody/10^8^ cells (B cells) and 0.25 μg biotinylated anti-Ter119 antibody/10^8^ cells (red blood cells) in 1 mL FACS buffer for 20 min on ice. Cells were washed and supernatant aspirated. They were then resuspended in MACS labelling buffer (2 mM EDTA/0.5% bovine serum albumin in PBS) at 10^8^ cells/mL and 20 μl of anti-biotin microbeads/10^8^ cells on ice for 25 min. Cells were washed twice in MACS buffer and resuspended in 500 μl of buffer prior to running cells through LS columns (Miltenyi) in a SuperMACS II Separation Unit (Miltenyi). T, B and red blood cells were removed via binding to microbeads in the LS columns. The column was washed thrice with MACS buffer and unbounded cells collected as flow-through.

CD8^+^ T cells were isolated from OT-I TCR-tg mice specific for ovalbumin (OVA)_257–264_/H-2K^b^ and CD4^+^ T cells were isolated from OT-II TCR-tg mice specific for IA^b^/OVA_323–339_, using MACS magnetic microbead separation technology as described above. CD8^+^ T cells were enriched from splenocytes via negative depletion of myeloid cells, granulocytes, DC, B cells and CD4^+^ T cells using specific antibodies: 0.25 μg biotinylated anti-CD19 antibody/10^8^ cells (B cells), 0.25 μg biotinylated anti-MHCII antibody/10^8^ cells (DC), 0.25 μg biotinylated anti-Gr1 antibody/10^8^ cells (granulocytes and myeloid cells) and 0.25 μg biotinylated anti-CD4 antibody/10^8^ cells. Similarly, CD4^+^ T cells were enriched by substituting antibody to deplete CD8^+^ T cells in the above cocktail: 0.25 μg biotinylated anti-CD8 antibody/10^8^ cells.

### Flow cytometry

Methods used for antibody staining and flow cytometry for analysis of cell surface marker expression have been described previously [10, 33, 34]. Prior to antibody staining, non-specific antibody binding to cells was inhibited by absorption of anti-CD16/32 (FcBlock: Biolegend: San Diego, CA, USA) used at 5 μg/10^6^ cells in 1 mL of FACS buffer. Fluorochrome- or biotin-conjugated antibodies specific for CD11c (N418), CD11b (M1/70), CD8 (53–6.7), CD19 (1D3), CD43 (IBII), F4/80 (CI:A3-1), Ter119 (Ter119), Thy1.2 (30-H12), Siglec-F (E50-2440), Ly6C (HK1.4), Ly6G (1A8) and I-A/I-E (M5/114.15.2) were purchased from Biolegend. Antibodies specific for CD68 (FA-11) and SIGN-R1 (ER-TR9) were purchased from AbD Serotec. Lastly, antibody specific for MOMA-1 (MOMA-1) was purchased from AbCam. Propidium iodide (PI) staining prior to flow cytometry was used to distinguish live and dead cells. Flow cytometry was performed on a BD LSRII flow cytometer (Becton Dickinson: Franklin Lakes, NJ, USA). Data were collected in terms of forward scatter, side scatter and multiple fluorescence channels. BD FACSDiva Software (Becton Dickinson) was used to acquire data and analysis post-acquisition employed FlowJo software (Tree Star: Ashland, OR, USA).

For sorting, cells were stained with fluorochrome-labelled antibodies and subsets identified as described in Hey et al. [10] and summarised in Table 1. All incubation and washing steps were performed in sodium azide-free FACS buffer. Sorted populations were collected in complete medium for use in functional assays.

**Table 1:** Phenotypic identification of myeloid and dendritic subsets in spleen

Subsets	Phenotype^a^
CD8^+^ cDC	CD11b^−^CD11c^hi^CD8^+^CD43^−^Ly6C^−^Ly6G^−^MHCII^+^
CD8^−^ cDC	CD11b^+^CD11c^hi^CD8^−^CD43^−^Ly6C^−^Ly6G^−^MHCII^+^
L-DC	CD11b^hi^CD11c^lo^CD8^−^CD43^+^Ly6C^−^Ly6G^−^MHCII^−^Siglec-F^−^
Resident monocytes	CD11b^hi^CD11c^lo^CD8^−^CD43^hi^Ly6C^+^Ly6G^−^MHCII^−^Siglec-F^−^
Inflammatory monocytes	CD11b^hi^CD11c^−^CD8^−^CD43^+^Ly6C^hi^Ly6G^−^MHCII^−^Siglec-F^−^
Eosinophils	CD11b^hi^CD11c^−^CD8^−^CD43^+^Ly6C^+^Ly6G^−^MHCII^−^Siglec-F^+^
Neutrophil	CD11b^hi^CD11c^−^CD8^−^CD43^+^Ly6C^+^Ly6G^+^MHCII^−^Siglec-F^−^

^a^ Splenocytes were prepared by removal of red blood cells and T and B lymphocytes, and then cells stained with cocktails of antibodies for separation of subsets and sorting using flow cytometry. The phenotype of myeloid subsets was delineated previously in Hey et al. [10]. The phenotype of cDC subsets was taken from Merad et al. [56]

### Endocytosis assay

The capacity of cells to take up antigen in vivo was assessed by measuring uptake of labelled antigen using flow cytometry. Ovalbumin conjugated to FITC (OVA-FITC) was delivered intravenously to mice at 1 mg/mouse at different time points, as described previously [30], with spleens collected at the same time (Fig. 2a). Mannan conjugated to FITC (mannan-FITC) was a new compound and so an initial time course study was conducted using 1 mg of mannan-FITC per mouse. A dose response was then conducted using 3 h as the time for maximum uptake (Fig. 2b). Splenocytes were prepared by red blood cell lysis and enrichment of T and B cells by depletion as described above. Cells were then stained with specific antibodies to identify subsets and determine uptake of labelled antigen via flow cytometry.

### Cell culture

Cells were cultured in Dulbecco’s Modified Eagle Medium supplemented with 22.2 mM D-glucose, 13 μM folic acid, 27 μM L-asparagine, 5.5 mM L-arganine HCL, 10% heat inactivated fetal calf serum (JRH Biosciences: Lenexa, Kansas, USA), 10 mM Hepes (JRH Biosciences), 2 mM L-glutamine (JRH Biosciences), 17.1 μM streptomycin, 100U penicillin and 50 μM 2-mercaptoethanol (BDH Ltd.: Poole, England) per litre of medium. For culture, cells were maintained in 5% CO_2_ in air with 97% humidity at 37 °C.

### T cell activation

The ability of sorted dendritic and myeloid cells to activate T cells was measured by their capacity to induce antigen-specific activation and proliferation of anti-OVA CD4^+^ T cells isolated from OT-II TCR-tg mice, or anti-OVA CD8^+^ T cells isolated from OT-I TCR-tg mice. Dendritic and myeloid subsets were sorted from splenocytes prepared from transgenic Act-mOVA mice. Sorted cells express OVA peptides on MHCI and MHCII molecules after in vivo uptake and clearance of dead cells in mice. Thus, cDC isolated from Act-mOVA mice can cross-present antigen to CD8^+^ T cells. Antigen presenting cells (APC) were sorted as described in Hey et al. [10] and summarised in Table 1. Cells were plated in diluting numbers in the presence or absence of lipopolysaccharide (LPS: 10 μg/mL), prior to addition of T cells. In order to measure proliferation, T cells were labelled with 5-(and 6-) carboxyfluorescein diacetate succinimidyl ester (CFSE: Molecular Probes: Eugene, Oregon, USA) as described previously [33]. T cells were labelled at a final concentration of 2.5 μM CFSE per 10^7^cells/mL in CFSE labelling buffer (PBS/0.1%BSA). T cells were cocultured with APC and collected after 72 h to determine T cell proliferation flow cytometrically by quantitation of CFSE staining. Proliferation was assessed in terms of dilution of fluorescent stain with each cell division.

### Measuring a cytotoxic T cell response in vivo

The ability of APC to induce a cytotoxic T lymphocyte response was investigated via lysis of target cells. Targeted cell lysis was measured using a fluorescent target array developed by Quah et al. [35]. The experimental protocol is summarised in Fig. 4a. On Day 0, CD8^+^ T cells were isolated from OT-I TCR-Tg mice. Equal numbers of CD8^+^ T cells were delivered into individual host mice (C57BL/6 J) via intravenous injection (3.5 × 10^6^ cells/mouse). At one hour after delivery of T cells, APC subsets sorted from Act-mOVA mice were also delivered into host mice. Three concentrations of APC were used: 90,000, 9000 and 900 cells. The effector function of activated CD8^+^ T cells was measured on Day 7 via lysis of peptide-pulsed target cells adoptively transferred intravenously on Day 6. Target cells were isolated from B6. SJL spleen and labelled with CFSE, Cell Trace Violet (Molecular Probes, Invitrogen) and Cell Proliferation Dye (eBioscience). Splenocytes were resuspended at 0.5-2 × 10^8^ cells/mL in 20 °C in medium and labelled with a final concentration of 0, 400, 2250 and 12,500nM of each dye. Labelled splenocytes were pulsed with SIIN (SIINFEKL), N6 (SIINFNKL), G4 (SIIGFEKL) and E1 (EIINFEKL), respectively, for an hour at 37 °C. All peptides were synthesised at the Biomolecular Research Facility (Australian National University). Pulsed target cells (2.5 × 10^7^) were delivered intravenously into host mice one day prior to harvesting host splenocytes for flow cytometric analysis of target cells. Flow cytometry was used to estimate the total number of target cells left in spleen. Specific killing of target cells was determined by the following formula which has been described previously [35].

$$ \%\kern0.5em \mathrm{specific}\kern0.5em \mathrm{lysis} = \left[1-\left(\frac{\raisebox{1ex}{${\mathrm{Targets}}_{\mathrm{primed}}^{+\mathrm{peptide}}$}\!\left/ \!\raisebox{-1ex}{${\mathrm{Targets}}_{\mathrm{primed}}^{+\mathrm{nil}}$}\right.}{\raisebox{1ex}{${\mathrm{Targets}}_{\mathrm{naive}}^{+\mathrm{peptide}}$}\!\left/ \!\raisebox{-1ex}{${\mathrm{Targets}}_{\mathrm{naive}}^{+\mathrm{nil}}$}\right.}\right)\ \right] \times 100 $$

### Statistical analysis

Data have been presented as mean ± standard error for sample size n. Where a normal distribution could be assumed, Students’ *t*-test was used to determine significance (*p* ≤ 0.05). For sample size *n* ≤ 5, where normal distribution cannot be assumed, the Wilcoxon Rank Sum test was used to test significant (*p* ≤ 0.05).

## Results

### Phenotypic identification of dendritic and myeloid subsets in spleen

A combination of cell surface markers was used to identify DC and myeloid subsets in spleen following a recent published procedure [10]. Conventional DC were gated as CD11c^hi^MHCII^+^ cells, then further delineated to give CD8^+^ cDC and CD8^−^ cDC on the basis of CD8 and CD11b expression (Table 1). Myeloid cells were initially gated as CD11b^hi^CD11c^−^ cells, then further delineated to give neutrophils, inflammatory monocytes and eosinophils on the basis of Ly6C, Ly6G and Siglec-F expression (Table 1). While L-DC and resident monocytes share a common CD11b^hi^CD11c^lo^ profile, they can be distinguished on the basis of Ly6C, Ly6G, CD43 and CX_3_CR1 expression (Table 1) [10].

### L-DC are distinct from splenic macrophages

**Fig. 1:**
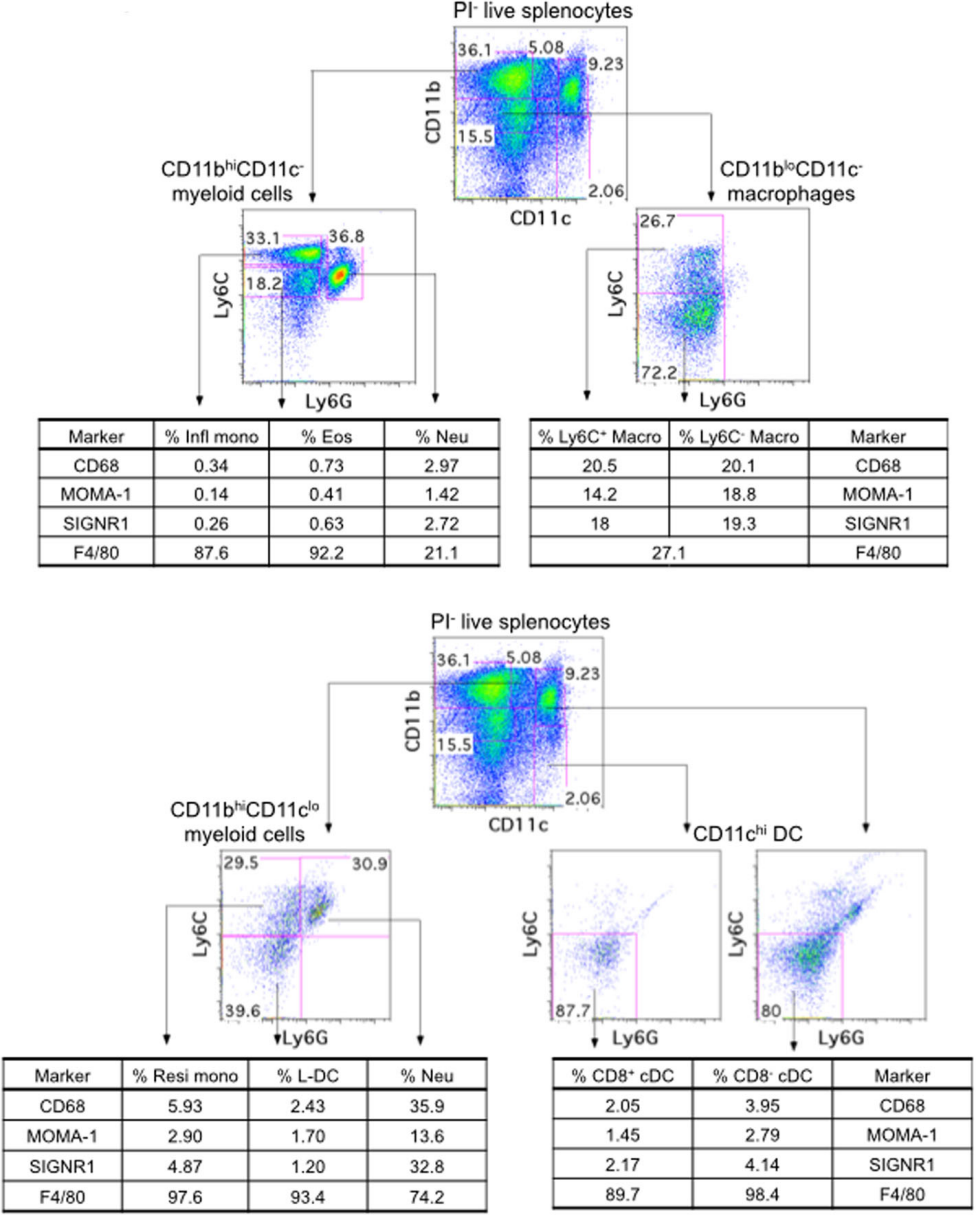
Expression of macrophage specific markers. Splenocytes were clear of red blood cells by lysis and enriched for myeloid and DC subsets via T and B cell depletion. Cells were then stained with fluorochrome-conjugated antibodies specific for CD11b (PE-Cy7), CD11c (APC), Ly6C (FITC), Ly6G (PE), along with biotinylated antibodies to CD68, MOMA-1, SIGNR1 and F4/80. APC-Cy7-streptavidin was used as a secondary conjugate. L-DC, dendritic and myeloid subsets were gated as described in Table 1 and Hey et al., (2016) [10]. **a** Expression of CD68, MOMA-1, SIGNR1 and F4/80 on inflammatory monocytes (Infl mono), eosinophils (Eos), neutrophils (Neu) and macrophages (Macro). **b** Expression of CD68, MOMA-1, SIGNR1 and F4/80 on resident monocytes (Resi mono), L-DC and cDC subsets. Data are reflective of three independent analyses

Macrophages have been historically characterised in spleen by immunohistological analysis. While some macrophage-specific markers have been identified, macrophage subsets are not well defined. Expression of markers like MOMA-1, SIGNR1, CD68 and F4/80 was therefore investigated on splenocytes. For each of the four macrophage markers, marker positive cells were initially gated and found to display a majority phenotype (40–50%) as CD11b^+^CD11c^−^Ly6C^+/−^Ly6G^−^ macrophages (data not shown). Subsequently, myeloid subsets and L-DC were gated as described in Table 1 and Hey et al. [10], and assessed for expression of specific macrophage markers (Fig. 1). Gated inflammatory monocytes, eosinophils and neutrophils did not stain for any of the macrophage markers, except F4/80. However, all gated myeloid and DC subsets stained for F4/80, and this is consistent with multiple studies which have demonstrated that F4/80 staining is not restricted to red pulp macrophages [36–38]. Neither resident monocytes, cDC nor L-DC showed expression of macrophage specific markers (Fig. 1).

### Antigen uptake capacity of L-DC compared with myeloid subsets

**Fig. 2:**
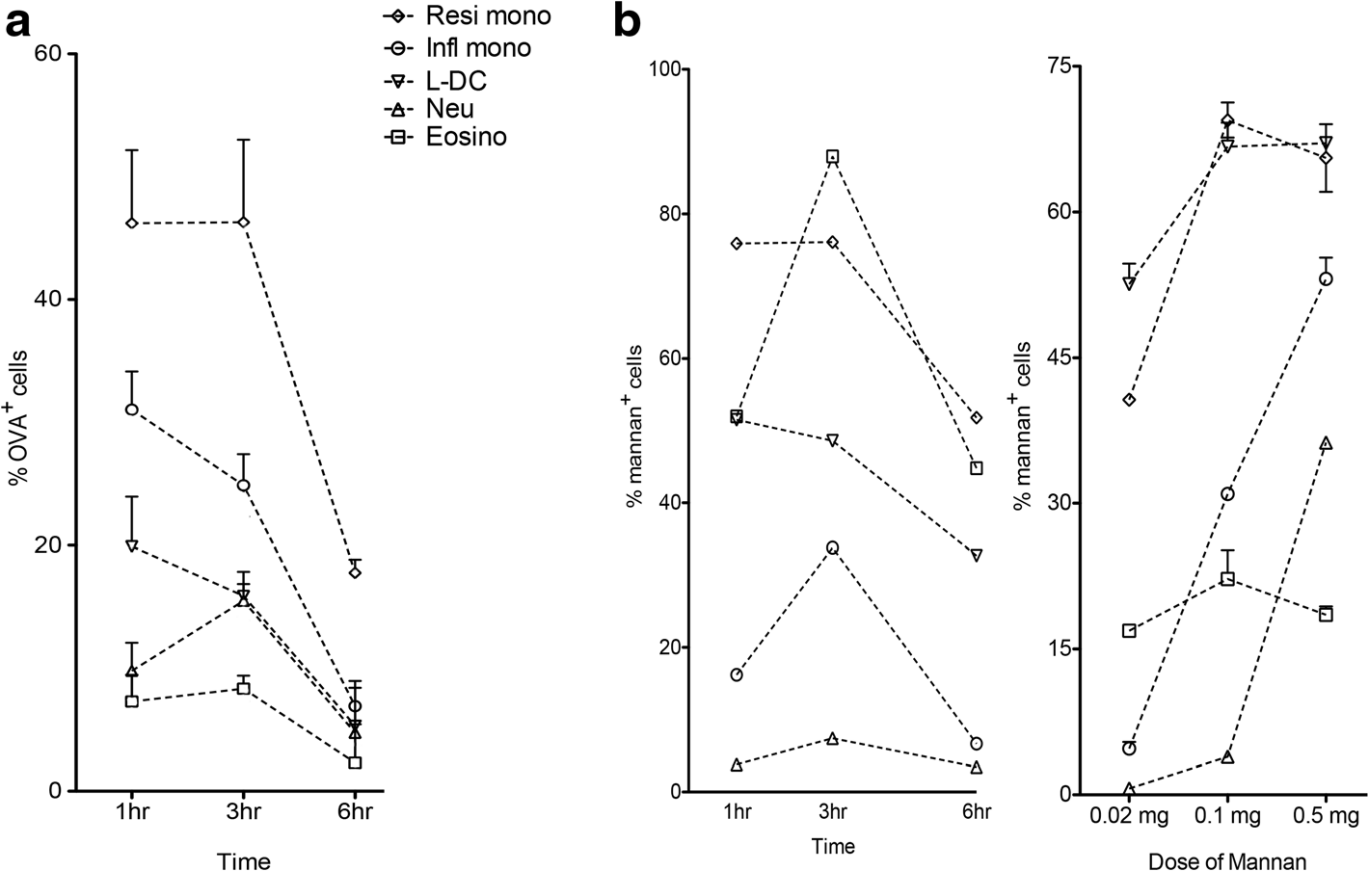
Comparison of endocytic ability of myeloid and dendritic subsets. The ability of cells to endocytose antigen was measured by uptake of OVA-FITC and mannan-FITC. Spleens were collected for analysis at the same time, and splenocytes prepared by lysis of red blood cells with enrichment for dendritic and myeloid cells via T and B cell depletion. Cells were stained with antibodies to identify L-DC and myeloid subsets as shown in Table 1. Uptake of antigen was assessed in terms of % FITC staining cells. C57BL/6 J mice were given: **a** OVA-FITC at 1, 3, and 6 h prior to euthanasia for spleen collection (intravenously; 1 mg per mouse). Data reflect mean ± SE (*n* = 4); **b** mannan-FITC (intravenously; 0.5 mg per mouse) at 1, 3 and 6 h prior to euthanasia for spleen collection. Single mice only were analysed tin a pilot study to determibe optimal time of 3 h used in a subsequent dose response experiment. Data reflect mean ± SE (*n* = 2). Control mice were given PBS

A primary function of spleen is to filter and trap blood-borne antigens, and this involves uptake and processing of antigen by dendritic and myeloid cells. Antigen presenting cells express a combination of receptors for uptake of antigen of different type via different pathways for endocytosis. In this study, pinocytosis and receptor-mediated endocytosis of antigen were investigated for the splenic myeloid subsets described in Table 1. FITC-labelled antigens were delivered intravenously into mice with subsequent isolation of subsets to compare uptake over time. Pinocytosis was studied by uptake of OVA-FITC as a soluble antigen under conditions described previously [30]. Amongst the splenic myeloid subsets, both resident and inflammatory monocytes showed ability to endocytose and retain OVA, with resident monocytes the most potent (Fig. 2a). Only ~10% of neutrophils and eosinophils took up and retained OVA (Fig. 2a). Their endocytic capacity was relatively weak compared with monocytes (Fig. 2a). L-DC displayed some uptake of OVA, but the level was relatively low compared with monocytes (Fig. 2a).

Mannose receptor-mediated uptake of antigen in DC has been found to contribute to cross-presentation of antigen to CD8^+^ T cells [39, 40]. Cross-presentation is a defining property of DC, and is clearly a property of splenic CD8^+^ cDC [23]. In order to determine if L-DC and other myeloid subsets can endocytose antigen via mannose receptors, mannan-FITC was prepared and delivered intravenously to mice and cell uptake monitored in a pilot study to determine optimal time for uptake. A further experiment using a 3 h time for uptake, then determined the minimum saturating dose of FITC-mannan as 0.1 mg per mouse. In the time course study, both resident and inflammatory monocytes demonstrated the strongest ability to take up mannan, with >75% uptake after 1 h, and >50% of resident monocytes retaining mannan after 6 h (Fig. 2b). In contrast, inflammatory monocytes displayed peak uptake of 88% at 3 h, with retention of antigen for 6 h by ~50% of cells (Fig. 2b). Delayed uptake by inflammatory monocytes, could reflect lower accessibility to antigen in comparison with resident monocytes. About 35% of eosinophils took up mannan by 3 h after delivery, but this diminished by 6 h (Fig. 2b). Neutrophils showed no endocytosis of mannan-FITC. Notably, L-DC showed high ability to take up and retain mannan although at lower levels than monocytes (~50%) (Fig. 2b). In the dose response experiment, both L-DC and resident monocytes gave strong early uptake, while inflammatory monocytes required infusion of more FITC-mannan to reach the same level of uptake (Fig. 2b). In an in vivo assay of this type, the level of mannan in cells is indicative of both the accessibility of cells to blood-borne antigen and the endocytic ability of cells. Resident monocytes took up and retained the highest level of mannan, followed by inflammatory monocytes, then L-DC (Fig. 2b).

### Ability of splenic myeloid and DC subsets to activate CD4^+^ T cells

A known property of antigen presenting DC and some macrophages is their ability to process exogenous antigen and present it as peptides on MHC-II molecules for CD4^+^ T cell activation. Previously it was shown that L-DC generated in vitro in long-term splenic cultures, or in stromal co-cultures, lack ability to activate CD4^+^ T cells, consistent with their absence of cell surface MHC-II expression [30, 33, 41]. Improved methodology for distinguishing dendritic and myeloid subsets in spleen now allows the question of the antigen presenting capacity of different subsets to be addressed with more certainty. The Act-mOVA mouse model was employed as a source of OVA antigen-expressing APC. Splenic myeloid and DC subsets were therefore sorted from Act-mOVA mice according to the criteria shown in Table 1, and compared for capacity to induce proliferation of CD4^+^ T cells isolated from OT-II TCR-tg anti-OVA mice.

**Fig. 3:**
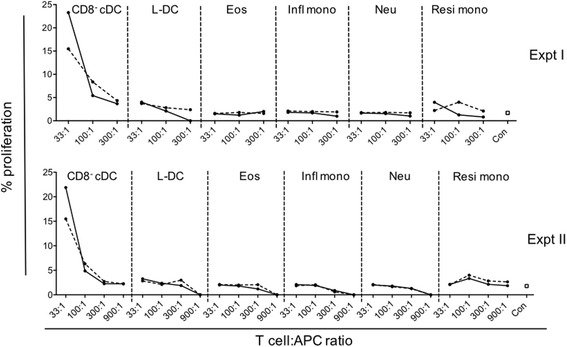
Activation of CD4^+^ T cells by splenic dendritic and myeloid subsets. Antigen presenting ability of myeloid subsets purified from spleens of Act-mOVA mice was assessed. L-DC, eosinophils (Eos), inflammatory monocytes (Infl mono), neutrophils (Neu), resident monocytes (Resi mono) and CD8^−^ cDC (as a control), were sorted as described in Table 1 following enrichment of splenocytes by depletion of red blood cells and T and B lymphocytes using magnetic bead technology. Diluting numbers of APC were plated following treatment with LPS (10 μg/ml) (solid line) and without LPS (dotted line) for 2 h. This was followed by addition of 10^5^ CFSE-labelled OT-II (TCR-tg) CD4^+^ T cells purified from mouse spleen through depletion of B cells, CD8^+^ T cells, DC and myeloid cells using magnetic bead protocols. Cells were cultured at T cell:APC ratios of 33:1, 100:1, 300:1 and 900:1 for 72 h. CD4^+^ OT-II T cells were then gated as PI^−^Thy1.2^+^Vα2^+^CD8^−^ cells, and assessed flow cytometrically for CFSE dilution as an indicator of T cell proliferation. OT-II T cells cultured alone served as controls (con). Graphs show % proliferating OT-II cells. Two independent replicate experiments were conducted

Consistent with the literature [42], CD8^−^ cDC were found to be very strong inducers of CD4^+^ T cell proliferation (Fig. 3). Neutrophils, inflammatory monocytes and eosinophils induced no CD4^+^ T cell proliferation. L-DC and resident monocytes induced no or very low levels of CD4^+^ T cell proliferation, equivalent to the control population of only T cells (Fig. 3). The addition of lipopolysaccharide (LPS) as a stimulator of APC did not improve the activation of CD4^+^ T cells, except in the case of CD8^−^ cDC where there was a minor increase at the highest T cell: APC ratio (Fig. 3). L-DC and monocyte subsets lacked ability to activate CD4^+^ T cells, despite their ability to endocytose and process mannan as an antigen, or OVA as a soluble antigen in the case of resident monocytes. Inability to activate CD4^+^ T cells is also consistent with lack of MHC-II expression by L-DC and the two monocyte subsets. These data serve to distinguish L-DC from professional APC like cDC.

### Can L-DC cross present antigen for CD8^+^ T cell activation?

Cross presentation appears to be a property of DC, and has been clearly demonstrated for the CD8α^+^ subset of cDC [42–44]. Early studies suggested that cross presentation was restricted to DC [45], although some macrophage and neutrophil subsets were found to have cross presenting ability under specific conditions [45–50]. The techniques used to isolate pure subsets of cells in those earlier studies were not as specific as those used here. The cross presenting ability of L-DC and the myeloid subsets listed in Table 1 has been assessed in relation to CD8^+^ cDC. Subsets were isolated from Act-mOVA mice and used to activate purified OTI (TCR-tg; anti-OVA) CD8^+^ T cells. T cells and APC were co-cultured for 72 h at ratios of 33, 100, 300 and 900:1 T cells:APC. The % proliferation of T cells was measured, and the ratio of T cell/APC required to induce 50% proliferation of CD8^+^ T cells used to compare data across replicate experiments (Table 2). The assay was performed in the presence and absence of LPS which can act as a potent inflammatory stimulus for some APC [51]. Consistent with previous reports in the literature, CD8^+^ cDC were superior in their ability to cross present antigen for CD8^+^ T cell activation and proliferation (Table 2), and this was shown over 6 independent experiments. In these experiments, neutrophils and inflammatory monocytes did not induce T cell proliferation through cross presentation of antigen. L-DC were up to 3-fold better activators of T cell proliferation than resident monocytes, but also 3-fold less effective than cDC in cross presentation of antigen for CD8^+^ T cell activation. In most experiments, the proliferation of T cells was not increased in the presence of LPS, suggesting it is not an activator of most of the APC subsets tested here. While outcomes from different experiments varied slightly, there was an overall trend showing that L-DC were better APC than resident monocytes, but were less effective than cDC. Inflammatory monocytes and neutrophils did not activate T cells.

**Table 2:** Cross priming capability of L-DC compared with myeloid cells

Expt	Condition	T cell: APC ratio giving 50% maximum proliferation of OT-I T cells^ab^				
		L-DC	Resi Mono	Infl mono	Neu	cDC
I	+ LPS	33	-	-	33	300
	- LPS	33	-	-	<33	300
II	+ LPS	33	33	-	-	300
	- LPS	33	<33	-	-	300
III	+ LPS	100	42	-	-	300
	- LPS	100	42	-	-	141
IV	+ LPS	100	42	<33	-	300
	- LPS	100	33	<33	-	300
V	- LPS	100	33	0	<33	-
VI	+ LPS	141	0	-	<33	-
	- LPS	100	0	-	<33	-

^a^For preparation of APC, splenocytes were harvested from Act-mOVA mice and prepared by red blood cell lysis followed by T and B cell depletion. L-DC, cDC, resident monocytes (Resi mono), inflammatory monocytes (Infl mono), neutrophils (Neu) were sorted as described in Table 1

^b^ APC, with and without LPS (10ug/ml), were cocultured with 10^5^ CFSE-labelled OT-I (TCR-tg) CD8^+^ T cells, sorted as PI^−^Thy1.2^+^Vα2^+^CD4^−^ cells, at T cell:APC ratios of 33, 100, 300 and 900:1. After 72 h, CD8^+^ OT-I T cells were gated as PI^−^CD11b^−^Thy1.2^+^Vα2^+^ cells, and CFSE dilution assessed flow cytometrically to estimate % proliferating T cells. OT-I T cells alone served as a control (Con)

### Induction of cytotoxic T cells by L-DC

**Fig. 4:**
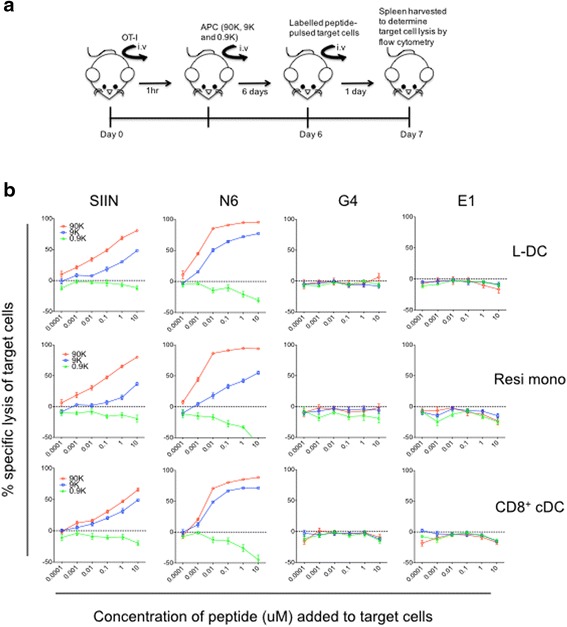
Ability of splenic APC to induce a cytotoxic T cell response. The ability of APC subsets to induce cytotoxic effector function in CD8^+^ T cells was assessed by measuring lysis of OVA peptide-pulsed target cells in a fluorescent target assay (FTA). **a** The experimental procedure is shown as a timeline. On Day 0, CD8^+^ T cells from OT-I TCR-Tg mice were prepared by red blood cell lysis of splenocytes and sorting for PI^−^Thy1.2^+^Vα2^+^CD4^−^ cells. OT-I T cells (3.5 × 10^6^) were delivered intravenously into host mice (C57BL/6). An hour later, several APC subsets sorted from Act-mOVA mice were also delivered into host mice. These were sorted as described in Table 1 and three cell doses (90 K, 9 K or 0.9 K) given intravenously. In order to measure the effector function of activated CD8^+^ T cells after 7 days, B6. SJL splenocytes were prepared as targets and adoptively transferred intravenously on Day 6. Target cells were labelled with several concentrations of CFSE, CTV and CPD for later identification. Overall, labelled target cells were then pulsed with 6 different concentrations of 4 distinct OVA peptides: SIINFEKL (SIIN), GLEQLESIINFEKL (N6), SIIGFEKL (G4) and EIINFEKL (E1). Specific killing of the distinctly labelled, antigen-pulsed target cells was determined by flow cytometric analysis to determine the number of target cells remaining in the test mouse compared with the control mouse given OT-I T cells only. Calculation of target lysis involved the formula described in Materials and Methods. **b** Data shows % specific lysis of target cells pulsed with different concentrations of peptides by OT-I T cells primed with three different APC types. Data is expressed as mean ± SE (*n* = 6)

The ability of APC to induce cytotoxic effector function along with proliferation of CD8^+^ T cells was tested by adoptive transfer in vivo. Experiments compared the ability of L-DC, resident monocytes and CD8^+^ cDC to induce cytotoxic effector function in CD8^+^ T cells using an in vivo cytotoxic killing assay developed by Quah et al. [35]. Sorted CD8^+^ OT-I (TCR-tg; anti-OVA) T cells were delivered intravenously into mice one hour ahead of APC sorted from ACTm-OVA mice as described in Table 1. Three doses of sorted APC were given to mice: 90,000, 9,000 and 900 cells. Six days later, labelled peptide-pulsed splenocytes were delivered intravenously to act as target cells for cytotoxic effectors primed by the sorted APC (Fig. 4a). At 1-day after delivery of target cells, host splenocytes were harvested to quantitate number of viable target cells in spleen to estimate % target cell lysis (Fig. 4). Ahead of adoptive transfer, target splenocytes were labelled with three different dyes used at four different concentrations. These were then pulsed with four different OVA peptides, either (SIIN) SIINFEKL, N6 (SIINFNKL), G4 (SIIGFEKL) or E1 (EIIFEKL), used at six different concentrations, so creating a multiplex assay. OT-I T cells can recognise the SIIN peptide but not the G4 and E1 peptides included as negative controls. The N6 peptide is a variant of SIIN peptide with phenylalanine and glutamic acid removed, which is also recognised by OT-I T cells. The response to N6 peptide determined in this in vivo response was of similar magnitude to that induced by the specific SIIN peptide. This could be a feature of this highly sensitive in vivo cytotoxic T cell assay [35].

The APC subsets of L-DC, resident monocytes and CD8^+^ cDC each induced cytotoxic T lymphocytes which then lysed target cells pulsed with SIIN and N6 peptides, but not target cells pulsed with the G4 or E1 peptides (Fig. 4b). In addition, the level of lysis of target cells reduced with decreasing concentration of peptides used to pulse APC, and the number of APC used to prime host mice (Fig. 4b). In order to directly compare APC subsets, the concentration of peptide required to prime cytotoxic T cells for 50% lysis of target cells was determined for each APC. With the N6 peptide, cytotoxic T cells generated by priming with 9,000 resident monocytes required 100 times more peptide to induce 50% lysis of target cells in comparison with L-DC and CD8^+^ cDC (Table 3). After priming with 90,000 APC, cytotoxic T lymphocytes generated using CD8^+^ cDC as APC, required 10 times more SIIN peptide to give 50% lysis of target cells in comparison with L-DC and resident monocytes (Table 3). When the number of priming APC was reduced to 9,000, cytotoxic T cells generated by resident monocytes required a higher concentration of SIIN to induce 50% lysis of target cells when compared with L-DC and CD8^+^ cDC (Table 3). Overall, resident monocytes were weaker inducers of a cytotoxic T lymphocyte response in comparison with L-DC and CD8^+^ cDC, which appear to induce similar cytotoxic effector responses.

**Table 3:** Capacity of splenic subset to induce a cytotoxic T cell response

Peptide	No. APC from Actm-OVA mice used for priming^a^ Targets:	Concentration of peptide (uM) required to activate CTL for 50% lysis of targets		
		L-DC	Resi Mono	CD8^+^cDC
SIIN	90 K	0.1	0.1	1.0
	9 K	10.0	> > 10	10.0
	0.9 K	0	0	0
N6	90 K	0.001	0.001	0.001
	9 K	0.01	1.0	0.01
	0.9 K	0	0	0
G4	90 K	0	0	0
	9 K	0	0	0
	0.9 K	0	0	0
E1	90 K	0	0	0
	9 K	0	0	0
	0.9 K	0	0	0

^a^ The table summarises data obtained from the fluorescent target experiment described in Fig. 3. Mice were given sorted OT-I CD8^+^ T cells ahead of APC sorted from Act-mOVA mice. These included L-DC, Resident monocytes (Resi mono) and CD8^+^ cDC. Target cells were added after 6 days, and their lysis measured in terms of % cell recovery at 24 h

## Discussion

Spleen plays a central role in immunity to blood-borne pathogens and cancer antigens. Ineffective immunity to bacteria at the level of spleen results in sepsis which can be both lethal and a costly complication of surgery and emergency medicine. Perisinusoidal niches housing hematopoietic stem cells were recently described in spleen red pulp [52], and this finding now opens fresh debate about the specific contribution of spleen to myelopoiesis and immunity. While the DC subsets in spleen have been well defined, other myeloid cells have not been systematically identified, with subset characterisation based only on the phenotype of similar cells in blood. With recent systematic identification of dendritic and myeloid subsets in spleen, and redefinition of monocytes and eosinophils [10], it is now possible to compare these subsets in terms of their capacity to uptake antigen, to activate CD4^+^ T cells, and to cross prime CD8^+^ T cells.

Several distinct dendritic and myeloid subsets were compared initially for capacity to endocytose antigen in vivo. While L-DC and resident monocytes have a similar CD11c^lo^ CD11b^hi^MHCII^−^ phenotype, differing in expression of Ly6C, CD43 and CX_3_CR1 (Table 1), they have very distinct antigen uptake abilities. Resident monocytes demonstrated superior ability to pinocytose soluble antigen in vivo, while L-DC were very weak, like eosinophils and neutrophils. However, L-DC and resident monocytes both showed high capacity for uptake of antigen by receptor-mediated endocytosis. Despite this similarity, only L-DC and not resident monocytes, could activate CD8^+^ T cells and induce cytotoxic effector cells. L-DC were also distinct from neutrophils which did not undergo receptor-mediated endocytosis or cross presentation. As shown previously by others, CD8^+^ cDC were highly endocytic of both soluble and particulate antigen, although resident and inflammatory monocytes demonstrated stronger receptor-mediated uptake. Previously, antigen uptake via mannose receptors was correlated with cross priming ability in DC [39, 40], although that study did not investigate the comparative ability of other myeloid subsets. Here we have been able to demonstrate that both resident and inflammatory monocyte subsets cannot cross prime CD8^+^ T cells, despite strong ability to take up antigen via mannose receptors. This suggests that cross presentation of antigen is unlinked to that antigen uptake pathway, and may require specific uptake or processing mechanisms.

One main aim of this project has been to compare L-DC with resident monocytes and cDC subsets in terms of capacity for T cell activation. A major limitation for in vitro studies of antigen presentation has been the low number of dendritic and myeloid cells present in spleen, and the difficulty of their isolation. Both CD8^+^ cDC and L-DC are very rare cell types, representing <1% of splenic leukocytes, and resident monocytes represent a 3-4-fold smaller population [10]. In order to overcome the limitation of low cell numbers, the Act-mOVA mouse model was used as a source of APC. These mice constitutively express high levels of cell-associated OVA under the actin promotor, and isolated APC express OVA peptides in association with MHCI and MHCII, without the need for antigen pulsing and washing of cells. In the animal, APC acquire OVA as an exogenous antigen through uptake of dead and dying cells then presented on MHCI and MHCII. However, when OVA is transcribed and expressed within the cell, there is also the possibility that OVA enters the endogenous antigen processing pathway whereby defective OVA is ubiquitin tagged for destruction in the cytoplasm. Since this type of processing could occur in all cells including APC, it has been necessary to use a control cell population as an indicator of endogenous cross presentation. In experiments described here, neutrophils which do not cross present antigen in the steady-state have served as an adequate control. In all experiments they induced little or no response in either CD8^+^ or CD4^+^ T cells.

Activation of CD4^+^ T cells is essential for induction of T helper cells, which then activate B cells and macrophages. Both CD8^+^ and CD8^−^ cDC induce activation of CD4^+^ T cells, although CD8^−^ cDC have been described as the strongest inducers [53]. Consistent with those findings, CD8^−^ cDC were shown to be strong activators of CD4^+^ T cells, while L-DC which lack MHCII expression show no capacity common to other MHCII^−^ myeloid cell types like eosinophils, neutrophils, inflammatory monocytes and resident monocytes. This is consistent with previous findings on L-DC produced in longterm spleen co-cultures, and in spleen stromal co-cultures [30, 33, 41].

While cross presentation is recognised as a characteristic property of DC, questions still remain as to the cross presenting capability of monocytes/macrophages and other myeloid subsets. Interpretation of published data is limited by the purity and certainty of subsets isolated for analysis, and of the conditions under which cells have been assessed. Indeed, a number of studies have described macrophages and neutrophils as able to cross present antigens to CD8^+^ T cells under inflammatory conditions. For example, it was recently shown that neutrophils from inflammatory peritoneal exudates could cross-prime CD8^+^ T cells both in vivo and in vitro [46, 54], While those studies demonstrated cross-priming by neutrophils, the described response occurred only under inflammation and so would not be reflective of neutrophils in steady-state spleen. Here two distinct assays have been used to analyse cross presenting ability, involving both in vitro and in vivo analyses, and measurement of a response in terms of both T cell proliferation and induction of T cell cytotoxicity. Both of these approaches have demonstrated the capacity of both cDC and L-DC to cross present antigen. Resident monocytes were quite distinctly different, at least 10-fold weaker then cDC, and 3- or 10-fold weaker than L-DC across two assays. Inflammatory monocytes and neutrophils were also incapable of cross presentation, and at least 10-fold weaker than cDC in inducing T cell proliferation. The treatment of all APC subsets with LPS did not improve activation of CD8^+^ T cells. Since L-DC were insensitive to LPS activation, it is unlikely that L-DC are monocyte-derived DC.

Cross priming for activation of CD8^+^ T cells is essential in the generation of cytotoxic T lymphocytes [55]. Cytotoxic T lymphocytes play an important dual functional role in maintaining self-tolerance by lysing self-targets and infected or cancerous cells. The ability of APC to cross present antigen and to induce cytotoxic T lymphocytes was investigated using a fluorescent target array which assesses cytotoxic T cell formation within the animal, where lytic activity against target cells is directly measured [35]. Cytotoxic T lymphocytes generated by OVA-expressing L-DC, lysed target cells pulsed with the SIIN and N6 peptides of OVA, but not with the G4 and E1 peptides. The cytotoxic T cell response induced was antigen-specific, since OT-I CD8^+^ T cells can only recognise SIIN and N6 peptides in the context of MHCI. Similarly, both CD8^+^ cDC and resident monocytes generated cytotoxic T lymphocytes which gave antigen-specific lysis of target cells when 9,000 APC were used, although at 90,000 APC, the activation capacity of CD8^+^ cDC was 10-fold lower. We attribute this to a saturation response in the presence of too many cells and too much peptide antigen. However, resident monocytes required a much higher concentration of peptide on target cells to give a similar level of lysis. Thus, resident monocytes are weaker inducers of cytotoxic T lymphocytes than both L-DC and CD8^+^ cDC, consistent with their lack of cross-presenting ability. These data serve to functionally distinguish the L-DC and resident monocyte subsets despite their similar but distinct phenotypes.

## Conclusion

This study identifies the distinct functional capacity of L-DC in terms of antigen presenting ability for CD4^+^ and CD8^+^ T cells. L-DC have been shown to be both phenotypically and functionally distinct from cDC subsets, resident and inflammatory monocytes, as well as neutrophils and eosinophils. They are not macrophages since they do not express markers which identify macrophage subsets unique to spleen. L-DC reflect a unique subset of cells resembling myeloid cells by phenotype, but a dendritic-like cell in terms of morphology, function in cross presentation, and ability to induce a cytotoxic T cell response.

## Abbreviations

APC: Antigen presenting cell
cDC: Conventional DC
CFSE: Carboxyfluorescein diacetate succinimidyl ester
DC: Dendritic cell
LPS: Lipopolysaccharide
OVA: Ovalbumin
pDC: Plasmacytoid DC
PI: Propidium iodide
TCR-tg: TCR transgenic
